# Rare case of *Ralstonia mannitolilytica* peritonitis in an adult peritoneal dialysis patient 

**DOI:** 10.5414/CNCS111202

**Published:** 2023-07-24

**Authors:** Joseph Kim, Litty Thomas, Kavita Bhavan, Ramesh Saxena

**Affiliations:** 1 Department of Internal Medicine Division of Nephrology, and; 2 Department of Internal Medicine Division of Infectious Disease, UT Southwestern Medical Center, Dallas, TX, USA

**Keywords:** peritoneal dialysis-associated peritonitis, PD, *Ralstonia mannitolilytica*

## Abstract

Peritonitis is a common complication of peritoneal dialysis (PD) usually caused by skin-dwelling Gram-positive bacteria and Gram-negative bacteria colonizing the gut and urinary tract. Occasionally, uncommon bacteria can cause peritonitis in PD patients. We describe a case of *Ralstonia mannitolilytica* peritonitis in a 67-year-old woman who has been on PD for more than 10 years with no prior episodes of peritonitis. To our knowledge, this is the first reported case of *Ralstonia* peritonitis in the United States. She initially presented with abdominal tenderness, right flank pain, and cloudy output from her nephrostomy tube. PD fluid and urine cultures grew *E. coli* which responded to treatment. However, her symptoms recurred after completion of antibiotic therapy with PD fluid growing *Ralstonia* species. She again responded to intraperitoneal antibiotics but had recurrence of symptoms after the completion of her second course of antibiotics. PD fluid grew *Ralstonia mannitolilytica* resistant to the prior antibiotic regimen. The PD catheter was removed, and she was transitioned to hemodialysis. Subsequent treatment led to the resolution of her symptoms. *Ralstonia* species are Gram-negative bacteria that are prevalent in water supplies and can form biofilms. They have been known to cause infection particularly in neonates, immunocompromised patients, or patients in intensive care. In our patient, prior antibiotic treatment for *E. coli* peritonitis is likely to have contributed to the development of *Ralstonia* peritonitis. Clinical improvement after removal of the PD catheter revealed that seeding from the PD catheter was the likely culprit for the recurrent infections.

## Introduction 

Despite improvement in overall rates in the past 20 years, peritoneal dialysis-associated peritonitis remains the leading complication of peritoneal dialysis (PD) and is associated with a high risk of morbidity and technique failure [1, 2, 3]. Most cases in PD patients are caused by common skin or nasal flora such as *Staphylococcus aureus* and Gram-negative bacteria such as *Pseudomonas, Klebsiella, Escherichia coli,* or *Enterobacter *[4]. *Ralstonia* species is an extremely rare cause of peritonitis. We describe a case of peritonitis caused by *Ralstonia mannitolilytica* in an adult PD patient. To our knowledge, this is the 1^st^ reported case of *Ralstonia mannitolilytica* peritonitis in an adult PD patient and the 5^th^ case overall for *Ralstonia* species infection in PD patients. 

## Case report 

Our patient is a 67-year-old woman with a history of end-stage kidney disease due to obstructive uropathy from renal tuberculosis and prior left nephrectomy. She has a right sided percutaneous nephrostomy (PCN) tube that is exchanged every 3 – 4 months and has had several episodes of urinary tract infections associated with these exchanges. She was subsequently started on fluoroquinolone prophylaxis prior to PCN exchange with no infection in the past 2 years. She has been on PD for 12 years with no prior history of peritonitis. 

The patient presented to the emergency room 1 day after a routine colonoscopy with severe abdominal pain, right-flank tenderness, and cloudy urinary output from the PCN. She was started on empiric antibiotic therapy with intravenous (IV) vancomycin and meropenem. Peritoneal fluid studies were consistent with peritonitis with a nucleated cell count of 2,364 cells/μL. Peritoneal fluid culture grew *E. coli* resistant to ciprofloxacin, levofloxacin, tetracyclines, and trimethoprim-sulfamethoxazole. Urine culture from the PCN also grew *E. coli*. She was transitioned to intraperitoneal (IP) cefazolin with subsequent clinical improvement (812 cells/μL at day 4 of treatment). She was discharged with a 3-week course of IP ceftazidime. 

A week after completing the antibiotics, the patient presented to the PD clinic with recurrent symptoms of abdominal pain. PD fluid cultures revealed Gram-negative rods with a cell count of 1,109 cells/μL. Empiric treatment was started with ceftazidime and vancomycin. Culture studies showed *Ralstonia* species sensitive to gentamicin, tobramycin, cefepime, ciprofloxacin, amikacin, levofloxacin and resistant to ceftazidime, piperacillin/tazobactam. She was transitioned to IP cefepime and gentamicin for 3 weeks. Follow-up visits after the antibiotic course showed clinical improvement. 

Two weeks after completion of the antibiotics, she presented to the PD clinic again with abdominal pain, nausea, vomiting and diarrhea. The peritoneal fluid analysis done in clinic confirmed a third episode of peritonitis with a cell count of 846 cells/μL. The patient was immediately admitted with repeat peritoneal fluid analysis revealing a cell count of 2,910 cells/μL. Culture studies revealed *Ralstonia mannitolilytica* resistant to ceftazidime, piperacillin/tazobactam, aztreonam, ceftriaxone, cefepime, meropenem, gentamicin, and tobramycin. Recurrent infection with the same organism after prior antibiotic treatment raised the concern of PD catheter seeding of the organism. Hence, the PD catheter was removed, and the patient was started on hemodialysis and oral trimethoprim/sulfamethoxazole based on the sensitivities with subsequent clinical improvement. 

## Discussion 

PD peritonitis is predominantly caused by *S. aureus* and other Gram-positive species and to a lesser extent by Gram-negative species [4]. *Ralstonia* species, as Gram-negative non-fermenters, are exceedingly rare causes of peritonitis. To our knowledge, there have been 3 documented cases of *Ralstonia pickettii* peritonitis in adult PD patients at a single UK nephrology center and only 1 case of *Ralstonia mannitolilytica* peritonitis reported in a pediatric PD patient from Greece [3]. Our case is the first documented report of *Ralstonia mannitolilytica* peritonitis in an adult PD patient (Table 1). 

*Ralstonia* species are a subset of Gram-negative bacteria that have become increasingly problematic in the healthcare setting. *Ralstonia* species can form biofilms which enhance the organisms’ survival and likely contributes to antibiotic resistance. As such, populations most vulnerable to *Ralstonia* species are those who are immunocompromised, in intensive care, have indwelling catheters, and neonates [5]. 

The *Ralstonia* species includes *Ralstonia pickettii, Ralstonia mannitolilytica, Ralstonia insidiosa, Ralstonia solanacearum, *and* Ralstonia syzygii*. *Ralstonia pickettii* and *Ralstonia mannitolilytica* have been commonly implicated in a wide variety of infections such as endocarditis, bacteremia, osteomyelitis, meningitis, and respiratory infections [5]. *Ralstonia mannitolilytica*, in particular, is known to be prevalent in water supplies and cause infection via sterile water contamination [2], catheter infections [6], hand contamination [3], and respiratory medical devices [7]. Distinguishing between the various *Ralstonia* species is difficult as they are not commonly monitored in the hospital setting and commercial identification systems are not equipped to distinguish between species. Polymerase chain reaction is currently the best way to identify the *Ralstonia* species [5]. 

In the 3 adult PD-associated *Ralstonia pickettii* cases, non-sterile glove use during the COVID-19 pandemic was a key factor in the outbreak at their nephrology center [8]. In the pediatric case of *Ralstonia mannitolilytica*, hand contamination was found to be the root cause of peritonitis [3]. The clinical course of our patient involved several peritonitis episodes and culminated in PD technique failure. Given that the *Ralstonia* peritonitis resolved only after PD catheter removal, we believe our patient’s PD catheter was seeded by *Ralstonia mannitolilytica*. There are potential reasons why our patient was vulnerable to peritonitis by this pathogen. While the other 4 cases of *Ralstonia* were associated with direct inoculation due to nonsterile technique, improper hygiene was unlikely to be the root cause of *Ralstonia* peritonitis in our patient who had no prior history of peritonitis. A more likely possibility is the disruption of the peritoneal microbiome through the administration of prior antibiotics leading to pathogenic proliferation of *Ralstonia mannitolilytica*. In the UK case series, one patient developed *Ralstonia* peritonitis immediately after being treated with antibiotics for *Corynebacterium* peritonitis [8]. While the peritoneal cavity has hitherto been considered sterile, there is emerging evidence that suggests that like gut, the peritoneum has its own microbiome [9]. *Ralstonia* has been documented to be a dominant genus in the peritoneal microbiota [10]. It is likely that a dynamic balance of resident microbes exists to maintain the microflora and protect against infections from pathogenic bacteria in the peritoneal environment. Alteration of the peritoneal microbiome by antibiotic treatments can dysregulate the peritoneal environment and contribute to the onset of infections in PD patients. 

In our patient, we observed a transition from *E. coli* to recurrent *Ralstonia mannitolilytica* peritonitis with rapid progression of antibiotic resistance where sensitivity was lost to cefepime and gentamicin. It is likely that prior use of antibiotics predisposed the patient to the initial *Ralstonia* peritonitis episode. The tendency of *Ralstonia* to form protective biofilms on the PD catheter also likely led to the development of antibiotic resistance resulting in recurrent infections. Once identified, removal of the catheter was crucial for the recovery of our patient. 

In summary, our case underlines the importance of rapid identification of PD catheter seeding and prompt removal of PD catheter with a targeted antibiotic regimen in the management of *Ralstonia* peritonitis. 

## Acknowledgment 

The authors would like to thank the nursing staff members of DaVita dialysis clinic and Clements University Hospital, Dallas, TX, USA who were involved in the management of this case and the Hirsch Family Foundation and the George M. O’Brien Kidney Research Core Center (P30DK079328) for their support during this case. 

## Funding 

This research received no specific grant from any funding agency in the public, commercial, or not-for-profit sectors. 

## Conflict of interest 

Ramesh Saxena received honorarium for speaker service from GSK. The other authors declare no relevant ﬁnancial interests. 

**Table 1.** Case comparison of each peritoneal dialysis-associated Ralstonia peritonitis.

	Case 1 (present case)	Case 2 [8]	Case 3 [8]	Case 4 [8]	Case 5 [3]
Age (years)	69	52	82	64	6
Gender	Female	Female	Female	Male	Female
Ethnicity	Asian	White	Asian	White	Not specified
Primary kidney disease	Obstructive uropathy secondary to renal tuberculosis	IgA nephropathy	Unknown	Diabetic nephropathy	Hemolytic uremic syndrome
Immunosuppression	No	Yes Prednisolone 5 mg daily	No	No	Not specified
PD catheter insertion time (months)	140	17	34	7	Not specified
Mode of PD	APD	APD	CAPD	APD	APD
History of Exit site infections	No	No	No	No	Not specified
Previous PD peritonitis	No	Yes	No	No	Yes
Clinical presentation	Abdominal pain, nausea, vomiting, cloudy PD fluid	Abdominal pain, cloudy PD fluid	Abdominal pain, cloudy PD fluid	Asymptomatic, pink PD fluid	Abdominal pain, cloudy PD fluid
*Species*	*Ralstonia mannitolilytica*	*Ralstonia pickettii*	*Ralstonia pickettii*	*Ralstonia pickettii*	*Ralstonia mannitolilytica*
Antibiotic treatment	1^st^ episode: empiric treatment into IP cefazolin and later ceftazidime. 2^nd^ episode: empiric treatment into IP cefepime and gentamicin. 3^rd^ episode: empiric treatment into oral trimethoprim/sulfamethoxazole	IP vancomycin into oral ciprofloxacin	IP gentamicin into IP vancomycin into oral ciprofloxacin	None	IP vancomycin and then IP ceftazidime
Clinical outcome	PD catheter was removed, and patient was transferred to hemodialysis with antibiotic treatment. Patient clinically improved.	Organisms cleared after antibiotic treatment. Patient clinically improved.	Organisms cleared after antibiotic treatment. Patient clinically improved.	Organisms cleared without antibiotic treatment.	Organisms cleared after antibiotic treatment. Patient clinically improved.

IP = intraperitoneal; PD = peritoneal dialysis; APD = automated peritoneal dialysis; CAPD = continuous ambulatory peritoneal dialysis.
